# The genetic and biochemical basis of trimethylaminuria in an Irish cohort

**DOI:** 10.1002/jmd2.12028

**Published:** 2019-03-25

**Authors:** Samantha Doyle, James J. O'Byrne, Mandy Nesbitt, Daniel N. Murphy, Zaza Abidin, Niall Byrne, Gregory Pastores, Richard Kirk, Eileen P. Treacy

**Affiliations:** ^1^ National Centre for Inherited Metabolic Disorders, The Mater Misericordiae University Hospital Dublin Ireland; ^2^ Sheffield Diagnostic Genetics Service, Sheffield Children's NHS Trust Sheffield UK; ^3^ National Rare Diseases Office, The Mater Misericordiae University Hospital Dublin Ireland; ^4^ School of Medicine and Medical Sciences, University College Dublin Dublin Ireland; ^5^ Department of Paediatrics, Trinity College Dublin, The University of Dublin Dublin Ireland

**Keywords:** fish odor syndrome, *FMO3* gene, genetic polymorphism, genotype, trimethylamine, trimethylamine *N*‐oxide

## Abstract

**Background:**

Inherited trimethylaminuria (TMAU), a rare genetic disorder of hepatic metabolism of trimethylamine (TMA) causing excessive accumulation of malodorous trimethylamine (TMA), is a socially distressing disorder. Diagnosis is made by biochemical analysis of urine, with the calculation of flavin monooxygenase trimethylamine conversion capacity. Genetic testing, sequencing the entire coding region of the *FMO3* gene has been recommended for affected individuals who convert less than 90% of the total TMA load to TMAO.

**Methods:**

Genetic analysis was undertaken for 13 Irish patients with TMAU of varying phenotypic severity (three severe, six moderate, and four mild).

**Results:**

A genetic diagnosis was made for seven patients, including for five of the nine moderate to severely affected cases. We noted the c.913G>T;p.(Glu305*) and c.458C>T;p.(Pro153Leu) mutations in this Irish population with severe TMAU which is consistent with our earlier findings in Australian and North American families of Irish and British descent.

Three individuals were noted to be homozygous for the common variant haplotype c.472G>A;923A>G;p.(Glu158Lys);(Glu308Gly). We also identified three novel variants in this population, which are likely to be pathogenic: c.682G>A;p(Gly228Ser), c.694G>T:p(Asp232Tyr), and c.989G>A;p.(Gly330Glu).

**Conclusion:**

Urinary biochemical analysis probably remains the first line diagnostic approach to classify the various types of TMAU. *FMO3* gene analysis is likely only to be informative for certain presentations of TMAU.

## 1 BACKGROUND

Inherited trimethylaminuria (TMAU; OMIM #602079) is a well‐described rare autosomal recessive genetic disorder associated with decreased hepatic trimethylamine *N*‐oxidation, which leads to an excess of the volatile trimethylamine (TMA) instead of substrate conversion to trimethylamine *N*‐oxide (TMAO).^1^, ^2^, ^3^ TMA is a tertiary amine derived from the enterobacterial metabolism of precursors such as choline and phosphatidylcholine present in the diet, and is also a bacterial metabolite of TMAO, a normal constituent of salt‐water fish. Most individuals convert >95% of the combined TMA/TMAO load to TMAO.^1^, ^4^ A diagnosis of TMAU is made using the measurement of TMA and TMAO in the urine.

TMAO/(TMA + TMAO) and is known as the FMO3 metabolic capacity. Based on these calculations, TMAU is classified as mild, moderate, or severe.

Rare loss of function pathogenic variants in flavin monooxygenase 3 (*FMO3*), the main FMO liver isoform, present in homozygous or compound heterozygous states are associated with severe primary TMAU.^4^

Microsomal FMOs are NADPH‐dependent flavoprotein enzymes that catalyze NADPH‐dependent monooxygenation of nucleophilic nitrogen, sulfur and phosphorus‐containing drugs, pesticides, xenobiotic, and endogenous amines. Of note, a number of recent studies have suggested that increased formation of oxidized metabolites through this FMO3 pathway may have a role in cardiovascular disease.^5^, ^6^

A number of *FMO3* variants resulting in TMAU are described in the *FMO3* disease specific LOVD database (https://databases.lovd.nl/shared/genes/FMO3). In addition to loss of function pathogenic variants three common *FMO3* variants, which affect FMO3 function by reducing metabolism of substrates are well described with varying prevalence in different ethnic populations.^7^, ^8^, ^9^ In a previous study we identified the prevalence of these common variants in a French‐Canadian and a large Irish cohort.^9^ In addition to severe TMAU, milder cases may be caused by heterozygosity for mild and severe loss of function mutations or by homozygosity for *FMO3* common variants in association with other exogenous effects on FMO3 function such as substrate overload, intake of dietary FMO3 inhibitors (such as indoles in brassica vegetables) or possibly by other modifiers of FMO function.

The exacerbations in females perimenstrually is also proposed to result from further decrease in FMO3 activity with hormonal effects.^10^ An acquired type of TMAU has also been reported in adults following a viral illness, which may indicate underlying renal or liver disease or viral hepatitis.^1^ A transient type of TMAU, sometimes observed in childhood, is related to common *FMO3* variants and FMO transition during ontogeny.^11^, ^12^

TMAU is a socially distressing disorder, which results in an odor similar to rotten or decaying fish due to excess TMA being excreted in sweat, urine, breath, and reproductive fluids.^2^ Individuals with this disorder may suffer from low self‐esteem and depression, and cases of attempted suicide have been reported.^1^, ^13^ Affected individuals frequently request information about familial recurrence risk, which poses challenges for risk calculation for cases of less severe TMAU cases particularly if associated with the common *FMO3* variants.

In this study, severe TMAU was defined as a TMA to TMAO conversion rate of ≤60% while mild and moderate TMAU was defined as a conversion rate between 80%‐90% and 60%‐80%, respectively. Currently, the Clinical Utility Gene Card for TMAU recommends *FMO3* sequencing analysis in individuals who convert less than 90% of the total TMA load to TMAO.^4^

The prevalence of primary TMAU is unknown although it is thought to be underdiagnosed. The estimated carrier frequency of *FMO3* pathogenic variants in a British Caucasian population is 0.5% to 1% although there may be significant variation in carrier status in different ethnic groups.^4^, ^14^

To date, the genetic profile of the Irish individuals affected with TMAU and the severity of their clinical expression has not been studied. We thus sought to determine the utility of gene sequencing vs standard metabolic/biochemical testing for TMAU in a cohort of adults who have self‐reported to our adult national metabolic clinic during a 10‐year period.

## 2 METHODS

### 2.1 Study population

The adult patients included in the study were self‐referred to our national reference center for possible trimethylaminuria.

### 2.2 Biochemical diagnosis

The biochemical diagnosis was established using the first urine analyzed for each patient taking a normal unrestricted diet (ie, no choline restriction) as per departmental guidelines. Female patients were advised not to provide the urine sample perimenstrually.

Urinary TMA and TMAO were measured directly and simultaneously using untreated urine samples. Urinary measurement of TMA (with TMAO by titanium chloride reduction) was achieved using alkalinized samples heated in a headspace auto‐sampler with analysis of gaseous TMA by gas chromatography‐mass spectrometry.^15^ FMO3 metabolic conversion capacity was calculated using the equation TMAO/ (TMA + TMAO) × 100. The biochemical diagnosis of TMAU was made by identifying decreased conversion of TMA to TMAO (<90%).

### 2.3 FMO3 analysis

Analysis of *FMO3* was performed for 13 biochemically confirmed TMAU Irish Caucasian adults (3 males and 10 females) by sequencing the entire coding region including all intron‐exon boundaries. Pathogenicity was determined using the ACMG (American College of Medical Genetics) guidelines.^16^ Appropriate pretest genetic counseling was performed and consent obtained in all cases.

## 3 RESULTS

Thirteen affected individuals (3 males and 10 females) were included in the cohort; all were of Irish descent. The age range at the time of the study was from 18 to 72 years. Three individuals had severe TMAU, six had moderate TMAU while the other four had mild TMAU (see Table 1). Five of the nine individuals within the moderate to severe category (TMA conversion less than 80%) were confirmed to be either homozygous or compound heterozygous for a known or presumed *FMO3* loss of function variant (see Table 1).

**Table 1:** Patient characteristics

Patient ID	Gender and age	TMA μmol/mmol creatinine	TMA‐*N*‐oxide μmol/mmol creatinine	TMA *N*‐oxide/(TMA + TMA) *N*‐oxide (%)	Severity	Ethnicity	Genotype
1	M, 68	25.5	75	75%	Moderate	Irish	No pathogenic variant detected. Heterozygous for c.472G>A;923A>G, p.(Glu158Lys;Glu308Gly) common variant haplotype
2	M, 31	24.8	38.2	60%	Severe	Irish	Compound heterozygous for c.458C>T,p.(Pro153Leu) and c.682G>A,p.(Gly228Ser)
3	F, 35	11.8	32.5	73%	Moderate	Irish	Compound heterozygous for c.458C>T,p.(Pro153Leu) and c.694G>T, p.(Asp232Tyr)
4	F, 48	29.7	80.7	73%	Moderate	Irish	No pathogenic variant detected
5	F, 28	23.3	53.9	70%	Moderate	Irish	No pathogenic variant detected
6	F, 65	9	69.5	83%	Mild	Irish	No pathogenic variant detected
7	F, 30	18.6	89.3	83%	Mild	Irish	No pathogenic variant detected. Heterozygous for c.472G>A:923A>G, p.(Glu158Lys);(Glu308Gly) common variant haplotype
8	F, 72	42.9	58.6	58%	Severe	Irish	Homozygous for c.472G>T;923A>G, p.(Glu158Lys);(Glu308Gly)
9	F, 37	23.3	53.9	70%	Moderate	Irish	No pathogenic variant detected
10	F, 13	25.8	33.5	56%	Severe	Irish	Homozygous for c.913G>T,p.(Glu305*)
11	F, 28	18.1	44.3	71%	Moderate	Irish	Compound heterozygous for c.458C>T;p.(Prol53Leu) and c.989G>A;p.(Gly330Glu)
12	M, 35	6.3	25.6	80%	Mild	Irish	Homozygous: c.472G>A;923A>G:p.(Glu158Lys);(Glu308Gly)
13	F, 46	11.2	110.6	91%	Mild	Irish	Homozygous: c.472G>A;923A>G;p.(Glu158Lys);(Glu308Gly)

Of the four mild TMAU cases, two were homozygous for the common variant haplotype c.472G>A;923A>G;p. (Glu158Lys);(Glu308Gly), one patient had no pathogenic variants identified, and another was heterozygous only for the c.472G>A;923A>G; p.(Glu158Lys);(Glu308Gly) variant haplotype.

### 3.1 Novel variants

Novel *FMO3* variants were identified in three families (see Table 1; family ID: 2, 3, and 11) but co‐segregation studies were only possible in one due to unavailability of the parents (Table 1; family ID: 2). In this case, the study confirmed the variants were on opposite alleles.

Individual ID 2 with severe TMAU has the well‐described pathogenic variant c.458C>T, p.(Pro153Leu) on one allele. The other allele, *in trans*, is a novel likely pathogenic variant c.682G>A; p(Gly228Ser) as this substitution affects a highly conserved amino acid position and in silico analysis supports a deleterious effect.

Individual ID 3 with moderate TMAU has one allele with the well‐described pathogenic variant c.458C>T, p.(Pro153Leu). The other allele has a likely pathogenic variant c.694G>T, p.(Asp232Tyr).

Individual ID 11 with moderate TMAU has a well‐described pathogenic variant c.458C>T;p.(Pro153Leu) and a likely pathogenic mutation, c.989G>A;p.(Gly330Glu).

The in silico analyses described above for these three variants indicating their deleterious nature included the following analyses: SIFT (sorting intolerant from tolerant), PolyPhen (polymorphism phenotyping), MutationTaster, MutationAssessor, and FATHMM (Functional Analysis through Hidden Markov Models). We also calculated the PROVEAN (Protein Variation Effect Analyzer) score, which evaluates whether an amino acid substitution has an impact on the biological function of the protein. See Table 2 for summary of this analysis.

**Table 2:** Functional prediction of novel variants

Mutation	Coordinates	Amino acid change	Sequence ontology	SIFT score	PolyPhen score	MutationTaster prediction	FATHMM score	MutationAssessor score	PROVEAN score
c.682G>A	chr1: 171079993	G/S	Missense variant	0 (damaging)	1 (probably damaging)	Disease causing	−0.31 (tolerated)	3.655 (high impact)	−5.566 (deleterious)
c.694G>T	chr1: 171080005	D/Y	Missense variant	0 (damaging)	1 (probably damaging)	Disease causing	0.23 (tolerated)	4.43 (high impact)	−7.192 (deleterious)
c.989G>A	chr1:171083308	G/E	Missense variant	0 (damaging)	1 (probably damaging)	Disease causing	−2.05 (damaging)	4.425 (high impact)	−7.252 (deleterious)

Although not been previously reported, c.989G>A has been identified in two other UK‐based TMAU affected individuals (personal communication, Sheffield Diagnostics Genetics Service). This variant affects a highly conserved amino acid position, and in silico analysis supports a deleterious effect. It is present at an extremely low allele frequency in population databases and gnomAD, and was detected *in trans* with a pathogenic variant. The c.989G>A variant has a FATHMM score which suggests it is damaging (see below) and has an extremely low reported allele frequency (3.298e‐05 in ExAC).

## 4 DISCUSSION

Severe TMAU is a socially distressing, rare genetic disorder, which is primarily treated with dietary restriction of precursors of TMA including choline, lecithin, and TMAO. Patients sometimes find the diet very restrictive and compliance is often suboptimal. In addition, adequate choline dietary intake is essential for neurological functioning and the development of the fetus and infant. Individuals with mild and moderate TMAU disease may respond well to dietary restrictions and to the use of acid soaps, while others with more severe disease usually require further treatment options, which include antibiotics to suppress intestinal production of TMA, and riboflavin, which maximizes the FMO activity.^3^, ^15^ Identification of individuals who require more extensive treatment from the outset is important to differentiate from individuals with milder phenotypes who may carry common variants. In addition, adequate pretest and post‐test genetic counseling is recommended for affected families.

The Clinical Utility Gene Card recommends that patients who excrete 10% or more free TMA should have *FMO3* sequence analysis.^4^ Our study has demonstrated a low diagnostic yield for subjects who have mild TMAU in contrast to the severely affected cohort.

Three individuals (two females and one male) were identified to be homozygous for the common variant haplotype c.472G>A;923A>G; p.(Glu158Lys;Glu308Gly). One of the patients had severe TMAU and the other two had the mild form. Explanations for the difference in phenotypes may range from possible differences in dietary TMA precursor intake, the effect of FMO3 inhibitors at the time of the study, or the existence of other significant environmental or gene influences on FMO3 activity.

In our previous Irish study where a possible association between the variant polymorphic haplotypes and idiopathic hypertension was considered, the allele distribution in a total of 2036 Irish individuals was studied.^9^ The allele frequency of the variant haplotype c.472G>A;923A>G, p.(Glu158Lys;Glu308Gly) was noted to approximate 0.2, inferring that 4% of the Irish population are homozygous for this variant. However, clearly 4% of the population does not require a choline restricted diet, the majority being asymptomatic. This contrasts with an allele frequency for the variant haplotype of 0.15 as recently noted in a US population and a lower frequency in a Sicilian and Sardinian healthy population.^14^ In the latter study, although urinary ESI‐MS/MS determination of TMA/TMAO ratios showed a considerable reduction in FMO3 activity, the subjects did not present with the clinical features of TMAU.

Recently, TMA and TMAO have been implicated in the pathogenesis of cardiovascular disease subsequent to TMAO being identified as an oxidative marker and potential biomarker in metabolic profiling of affected cases.^6^ Whether TMAO is a surrogate marker of oxidative damage associated with other FMO3 mediated oxidized toxins remains to be determined. However, as with other polymorphisms of drug and toxin metabolizing genes, there is possibly a biological advantage for the evolutionarily conserved variants (Glu158Lys) and (Glu308Gly).^6^, ^8^, ^17^

A moderately affected individual was noted only to be a heterozygote for the c.472G>T; 923A>G (p.Glu158Lys;Glu308Gly) variant haplotype. In addition, no pathogenic variant was identified in four mild‐moderate patients. These cases may reflect the limitations in the *FMO3* analysis. Undetected variants in the deep introns (identifiable by RNA sequencing methods), or in the regulatory element of *FMO3*, deletion/duplications or balanced structural rearrangements involving *FMO3* which were not tested for could account for the disease in these cases.^18^ Although these investigations were not examined as part of this study, we suggest that such analysis should be considered for unresolved cases.

Another possibility is alternative diagnosis known to cause fish‐like odor or halitosis, such as dimethylglycinuria or SELENBP1 deficiency.^19^, ^20^ In addition, in a recent study, of 10 individuals presenting with impaired TMA oxidation, five were identified to have novel rare single nucleotide polymorphisms on other oxidoreductase genes, which may have a predicted effect on FMO3 function.^21^

The variants c.913G>T;p.(Glu 305*) and c.458C>T;p.(Pro153Leu) in this Irish population with severe TMAU is consistent with our earlier observations in Australian and North American families of Irish and British descent.^22^, ^23^ In addition to these two more commonly described *FMO3* variants and common polymorphisms other variants identified in cohort appear to be “private” variants as described in other populations.

## 5 CONCLUSION

This study illustrates that *FMO3* analysis is sensitive in diagnosing patients with severe TMAU. The use of *FMO3* analysis in the clinical management of this disorder may also be of benefit in the diagnosis of certain cases of intermittent or mild‐moderate TMAU.

We consider that biochemical diagnosis should remain the first line and main testing approach for individuals with suspected TMAU.

The polymorphic haplotypes are prevalent in the population with undetermined penetrance. As the majority of individuals carrying these common variants are likely to be asymptomatic, it may not be beneficial to identify *FMO3* variants, which results in mild TMAU, which could lead to detrimental over‐restriction of choline and other self‐imposed dietary imbalances.

## INFORMED CONSENT

All procedures followed were in accordance with the ethical standards of the responsible committee on human experimentation (institutional and national) and with the Helsinki Declaration of 1975, as revised in 2000 (5). Informed consent was obtained from all patients for being included in the study.

## AUTHOR CONTRIBUTIONS

S.D. carried out the patient reviews, obtained informed consent, delivered genetic counseling, wrote the paper, and made the final corrections. J.J.O. and G.P. reviewed the manuscript. M.N. analyzed the genetic samples and reported on the results along with reviewing the manuscript. D.N.M. provided bioinformatics support for the research. Z.A. helped with data collection. N.B. reviewed the database and helped to identify patients. R.K. reviewed the genetic results and reviewed the manuscript. E.P.T. conceptualized and supervised the research along with reviewing all submissions and providing expertise to the manuscript.

## References

[1] Treacy E . Trimethylaminuria and Deficiency of Flavin‐Containing Monooxygenase Type 3 (FMO3). The Metabolic and Molecular Bases of Inherited Disease (OMMBID). New York: McGraw‐Hill; 2007.

[2] Mackay RJ , McEntyre CJ , Henderson C , Lever M , George PM . Trimethylaminuria: causes and diagnosis of a socially distressing condition. Clin Biochem Rev. 2011;32(1):33. 21451776 PMC3052392

[3] Phillips IR , Shephard EA . Primary Trimethylaminuria. Gene Reviews—NCBI Bookshelf. https://www.ncbi.nlm.nih.gov/books/NBK1103/. [last accessed December 1, 2018]

[4] Shephard EA , Treacy EP , Phillips IR . Clinical utility gene card for: trimethylaminuria—update 2014. Eur J Hum Genet. 2015;23(9). https://doi.10.1038/ejhg.226. 10.1038/ejhg.2014.226 PMC4538216 25335494

[5] Li XS , Obeid S , Klingenberg R , et al. Gut microbiota‐dependent trimethylamine *N*‐oxide in acute coronary syndromes: a prognostic marker for incident cardiovascular events beyond traditional risk factors. Eur Heart J. 2017;38(11):814‐824. 28077467 10.1093/eurheartj/ehw582 PMC5837488

[6] Nowiński A , Ufnal M . Trimethylamine *N*‐oxide: a harmful, protective or diagnostic marker in lifestyle diseases? Nutrition. 2018;46:7‐12. 10.1016/j.nut.2017.08.001. 29290360

[7] Lambert DM , Mamer OA , Akerman BR , et al. In vivo variability of TMA oxidation is partially mediated by polymorphisms of the FMO3 gene. Mol Genet Metab. 2001;73(3):224‐229. 11461189 10.1006/mgme.2001.3189

[8] Phillips IR , Francois AA , Shephard EA . The flavin‐containing monoooxygenases (FMOs): genetic variation and its consequences for the metabolism of therapeutic drugs. Curr Pharmacogenomics. 2007;5(4):292‐313.

[9] Dolan C , Shields DC , Stanton A , et al. Polymorphisms of the flavin containing monooxygenase 3 (FMO3) gene do not predispose to essential hypertension in Caucasians. BMC Med Genet. 2005;6(1):41. 16324215 10.1186/1471-2350-6-41 PMC1316875

[10] Shimizu M , Cashman JR , Yamazaki H . Transient trimethylaminuria related to menstruation. BMC Med Genet. 2007;8(1):2. 17257434 10.1186/1471-2350-8-2 PMC1790885

[11] Koukouritaki SB , Simpson P , Yeung CK , Rettie AE , Hines RN . Human hepatic flavin‐containing monooxygenases 1 (FMO1) and 3 (FMO3) developmental expression. Pediatr Res. 2002;51(2):236‐243. 11809920 10.1203/00006450-200202000-00018

[12] Zschocke J , Kohlmueller D , Quak E , Meissner T , Hoffmann G , Mayatepek E . Mild trimethylaminuria caused by common variants in FM03 gene. Lancet. 1999;354(9181):834‐835. 10485731 10.1016/s0140-6736(99)80019-1

[13] Khan SA , Shagufta K . A rare case of fish odor syndrome presenting as depression. Indian J Psychiatry. 2014;56(2):185‐187. 24891709 10.4103/0019-5545.130505 PMC4040069

[14] D'Angelo R , Esposito T , Calabrò M , et al. FMO3 allelic variants in Sicilian and Sardinian populations: trimethylaminuria and absence of fish‐like body odor. Gene. 2013;515(2):410‐415. 23266626 10.1016/j.gene.2012.12.047

[15] Manning NJ , Allen EK , Kirk RJ , Sharrard MJ , Smith EJ . Riboflavin‐responsive trimethylaminuria in a patient with homocystinuria on betaine therapy. JIMD Rep. 2012;5:71‐75. 23430919 10.1007/8904_2011_99 PMC3509925

[16] Richards S , Aziz N , Bale S , et al. Standards and guidelines for the interpretation of sequence variants: a joint consensus recommendation of the American College of Medical Genetics and Genomics and the Association for Molecular Pathology. Genet Med. 2015;17(5):405‐423. 25741868 10.1038/gim.2015.30 PMC4544753

[17] Nebert DW . Polymorphisms in drug‐metabolising enzymes. What is their clinical relevance and why do they exist? Am J Hum Gen. 1997;60(2):265. PMC1712416 9012398

[18] Shimizu M , Allerston CK , Shephard EA , Phillips IR . Relationships between flavin mono‐oxygenase 3 (FMO3) genotype and trimethylaminuria phenotype in a Japanese population. Br J Clin Pharmacol. 2014;77(5):839‐851. 24028545 10.1111/bcp.12240 PMC4004404

[19] Moolenaar SH , Poggi‐Bach J , Engelke UF , et al. Defect in dimethylglycine dehydrogenase, a new inborn error of metabolism: NMR spectroscopy study. Clin Chem. 1999;45(4):459‐464. 10102904

[20] Pol A , Renkema GH , Tangerman A , et al. Mutations in SELENBP1, encoding a novel human methanethiol oxidase, cause extraoral halitosis. Nat Genet. 2018;50(1):120‐129. 29255262 10.1038/s41588-017-0006-7 PMC5742538

[21] Guo Y , Hwanf LD , Li J , et al. Genetic analysis of impaired trimethylamine metabolism using whole exome sequencing. BMC Med Genet. 2017;18(1):11 10.1186/s12881-017-0369-8. 28196478 PMC5310055

[22] Treacy EP , Akerman BR , Chow LM , et al. Mutations of the flavin‐containing monooxygenase gene (FMO3) cause trimethylaminuria, a defect in detoxication. Hum Mol Genet. 1998;7(5):839‐845. 9536088 10.1093/hmg/7.5.839

[23] Akerman B , Lemass H , Chow L , et al. Trimethylaminuria is caused by mutations of the FMO3 gene in a North American cohort. Mol Genet Metab. 1999;68(1):24‐31. 10479479 10.1006/mgme.1999.2885

